# Versatility of Cyclophilins in Plant Growth and Survival: A Case Study in *Arabidopsis*

**DOI:** 10.3390/biom9010020

**Published:** 2019-01-10

**Authors:** Izailda Barbosa dos Santos, Sang-Wook Park

**Affiliations:** Department of Entomology and Plant Pathology Auburn University, Auburn, AL 36849, USA; IBD0001@auburn.edu

**Keywords:** Cyclophilin, PPlases, *Arabidopsis*, organogenesis, defense responses

## Abstract

Cyclophilins (CYPs) belong to a peptidyl-prolyl *cis-trans* isomerase family, and were first characterized in mammals as a target of an immunosuppressive drug, cyclosporin A, preventing proinflammatory cytokine production. In *Arabidopsis*, 29 CYPs and CYP-like proteins are found across all subcellular compartments, involved in various physiological processes including transcriptional regulation, organogenesis, photosynthetic and hormone signaling pathways, stress adaptation and defense responses. These important but diverse activities of CYPs must be reflected by their versatility as cellular and molecular modulators. However, our current knowledge regarding their mode of actions is still far from complete. This review will briefly revisit recent progresses on the roles and mechanisms of CYPs in *Arabidopsis* studies, and information gaps within, which help understanding the phenotypic and environmental plasticity of plants.

## 1. Introduction

Cyclophilins (CYPs) are members of, namely, immunophilins that possess binding abilities towards immunosuppressive drugs such as cyclosporine A (CsA), FK506 and rapamycin. CsA binds to a group of CYP proteins, and FK506 and rapamycin bind to a distinct set of receptors, called FKBPs (FK506 and rapamycin-binding proteins), of which complexes (CsA-CYPs or FK506-FKBPs) inhibit nuclear translocation of NF-AT (nuclear factor of activated T-cells), and prevent the release of a proinflammatory cytokine, interleukin-2, and subsequent activation of immune responses, engendering immunosuppressive effects. Both CYPs and FKBPs exhibit a characteristic peptidyl-prolyl *cis-trans* isomerase (PPlase) activity which catalyzes the rotation of X-Pro peptide bonds from a *cis* to *trans* conformation, a rate-limiting step in protein folding or the assembly of protein complexes, tuning the roles and activities of a wide variety of proteins containing *trans*-prolyl imide bonds [1,2]. Note that *cis*-prolyl bonds are uncommon, most likely because of unfavorable contacts between adjacent amino acid residues in this isomeric form [3]. These post-translational modifications in turn coordinate a layer of primary and secondary metabolic pathways in diverse cellular processes. Hence, alteration of immunophilins’ functions and expressions render not only the loss of innate immunity, but also various diseases such as cancer, neurodegeneration, diabetes, asthma, rheumatoid arthritis, and cardiovascular, Parkinson’s and Alzheimer’s diseases, urging us to revisit their potential importance as drugs targets and pharmacological uses [1,2,3,4,5].

CYPs are structurally and evolutionally conserved PPIases found in all types of life including mammals, plants, insects, fungi and bacteria. They are categorized as single- and multi-domain PPlases; single-domain CYPs encode only a catalytic (PPlase) domain, referred to as CYP-like domain (CLD), whereas multi-domain CYPs include additional domains—in general—involved in protein and protein, or protein and nucleic acid interactions such as WD40 repeat, tetratricopeptide repeat, U-box, RNA recognition motif, Zn-finger, α-helical bundle, Leu-zipper, Ser/Lys and/or Arg/Glu-rich domains [1,2,6,7]. Reportedly, all CLD shares a common folding architecture consisting of eight antiparallel β-sheets, capped by three α-helices [1,2,3,6]. The second α-helix, placed between the β6 and β7 loop region, possesses an active site residue, Trp; the most conserved and critical amino acid (aa) for both catalytic and substrate/inhibitor binding activities. Shifting of the Trp to Ala or Phe showed a negative impact on PPlase activity and CsA-binding affinity [1,7,8,9,10]. In addition, three catalytic aa residues (Arg, Phe and His) are found across β3, β4 and β7 sheets which form a so-called ‘active pocket’ and facilitate the substrate bindings and metabolisms [1,7,11,12].

In plants, CYPs were first isolated in 1990 concomitantly from tomato (*Lycopersicon esculentum*), maize (*Zea mays*) and oilseed rape (*Brassica napus*) [13]. Since then, major efforts have been made to identify and characterize CYPs from *Arabidopsis*, a model plant system (designated as AtCYPs) [6,14,15,16,17,18,19,20]. In particular, two pioneering studies carrying out the comprehensive analyses of *Arabidopsis* genomics databases revealed 29 AtCYPs and CYP-like proteins. The surprisingly large number of AtCYPs along with their ubiquitous localizations across all subcellular compartments and widespread expressions throughout all major organs (e.g., flowers, leaves, stems and roots; except a specific expression of AtCYP26-1 in flowers) proposed that CYPs’ activities must be intrinsic in the growth and survival of *Arabidopsis* [1,7,21]. In agreement, several studies have unveiled the putative substrates, interacting partners, as well as biochemical and physiological activities of AtCYPs, corroborating the multifaceted roles of AtCYPs in broad ranges of cellular processes including transcriptional regulation, organogenesis, photosynthetic and hormone signaling pathways, stress adaptation and defense responses [22]. Now, this review will revisit the recent advances and working models of the functional circuitry of AtCYPs, and information gaps within, in effort to further understand the versatile activities of plant CYPs, and help delineating the phenotypic and environmental plasticity of plants.

## 2. Activities of Cyclophilins in Plant Growth and Development

**Figure 1:**
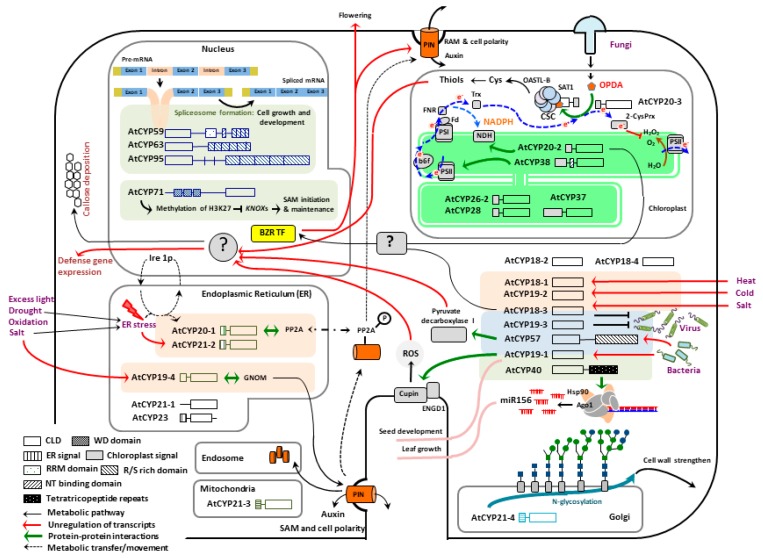
A working model; the metabolic and signaling pathways of AtCYPs in plant growth and defense responses. See the context for detailed explanation. Abbreviations: Ago1 (ARGONAUTE 1), BZR TF (BRASSINAZOLE RESISTNAT1 transcription factor), B_6_f (Cytochrome b_6_f complex), CSC (Cysteine synthase complex), Cupin (RmlC-like cupin superfamily), Cys (cysteine), ENGD1 (GTPase/GTP-binding protein), Fd (Ferredoxin), FNR (Ferredoxin NADPH reductase), GNOM (ADP ribosylation factor-guanine-nucleotide exchange factor), Hsp90 (Heat shock protein 90), H3K27 (Lysine 27 on histone H3 protein), Ire 1p (Inositol-requiring enzyme-1), KNOXs (KNOTTED-like homeoboxes), MiR156 (MicroRNA 156), NADPH (Nicotinamide adenine dinucleotide phosphate), NDH (NAD(P)H dehydrogenase), OASTL-B (*O*-actylserine(thiol)lyase B), OPDA [(+)-12-oxo-phytodienoic acid], P (Phosphorus), PIN (Pin-formed 1 auxin efflux carrier proteins), PP2A (Ser/Thr Protein Phosphatase 2), PSI & II (Photosystem I & II), RAM (Root apical meristem), ROS (Reactive oxygen species), SAM (Shot apical meristem), SAT1 (Serine actyltransferase1), Trx (Thioredoxin), and 2-CysPrx (2-Cys peroxiredoxin).

Recently, emerging evidences have elucidated that CYPs are important regulators in various metabolic pathways controlling organellar housekeeping, temporal and spatial specific metabolisms, as well as organismal development and growth in plants (Figure 1) [1,7,22]. These roles and activities of CYPs must be closely associated with their subcellular localizations. Especially, most CYPs share the same enzymatic (PPlase) activity and inhibitor (CsA), highlighting that their locations are the key limit factors of accessible substrates and interacting partners which in turn reflect their cellular activities and functions. This chapter thus will discuss recent advances in our understanding of CYPs in *Arabidopsis* growth and developmental processes in comparison with their subcellular locations.

### 2.1. Nuclear Localized AtCYPs

In *Arabidopsis*, four multi-domain AtCYPs (i.e., AtCYP59, AtCYP63, AtCYP71 and AtCYP95) were predicted to target the nucleus [1,7]. Among these, AtCYP63 and AtCYP95 harbor a C-terminal RS (Arg-Ser) rich domain, known to regulate protein and protein interactions in the formation of the spliceosomal complex and the activation of RNA polymerase II [1,23,24,25,26,27], suggesting their potential activities in RNA metabolisms [1]. In fact, their human counterparts such as SR-CYP, Matrin-CYP and hCYPH actually demonstrated binding affinity to a splicesomal snPNP complex and/or RNA polymerase II [28,29,30].

AtCYP59 is another nuclear AtCYP that contains a C-terminus RS rich domain, along with an N-terminal CLD, an RNA recognition motif and a zinc finger domain. The RS rich domain enables AtCYP59 to interact with a number of SR proteins (e.g., SR28, SR33 and SR35) involved in RNA splicing during various plant growth and developmental processes [31,32]. However, AtCYP59 appeared not to colocalize with those SR proteins in nuclear speckles, instead it showed a punctuate localization pattern resembling transcription initiation sites. In line with this scenario, in vitro protein and protein interaction assays exhibited the binding affinity of AtCYP59 to the nascent transcript of mRNA, as well as the C-terminal domain (CTD) of RNA polymerase II that is a binding platform of the transcription and splicing factors, and the nascent transcripts [31,33,34,35]. Perhaps, PPIase activity of AtCYP59 can modulate the structure and phosphorylation states of CTD, which in turn controls the transcription of selective mRNA associated with cell growth and development [31]. However, mRNA-binding spontaneously inhibits the PPlase activity of AtCYP59 [35], further suggesting that AtCYP59 is positioned at the interface of splicing and transcription, perhaps tuning the elongation of RNA polymerase, from where they might translocate to the nascent transcripts to ensure efficient splicing, concomitant with transcription [28,35].

AtCYP71, a highly conserved eukaryotic CYP, is an important regulator of organogenesis in *Arabidopsis*. Disruption of *AtCYP71* mRNA hence demonstrated the drastic disfiguration of the shape and size of leave, as well as petioles, via upregulating the expressions of a class I *KNOTTED-like* homeobox (*KNOX*) gene family including *SHOOT MERISTERMLESS* (*STM*), *KNOTTED-1-LIKE 1*/*2* (*KNAT1*/*2*) and *ASYMMETRIC LEAVES 1*/*2* (*AS1*/*2*), which is required for the initiation and maintenance of the shot apical meristem (SAM) [36,37]. These results suggest that AtCYP71 regulates negatively, or fine-tunes the expression of *KNOX* family genes. Interestingly, an N-terminus region of AtCYP71 possesses WD40 repeat domains, interacting with histone H3, chromatin assembly factor-1 and like-heterochromatin protein1, suggesting a potential role of AtCYP71 in the chromatin remodeling [37,38]. Histone modifications (i.e., methylation and acetylation) are lined to transcriptional controls. In particular, methylation of Lys^4^ in H3 is associated with activation of gene expressions, whereas methylation of Lys^9^ or Lys^27^ in H3 (H3K9/27) leads to gene silencing. In line with this scenario, AtCYP71 can binds and reinforces the methylation of H3K27 in the coding regions of e.g.,) *STM* and *KNAT1*, thereby maintaining the silenced state of those genes, and regulating proper SAM development in *Arabidopsis* [37].

### 2.2. Cytosol Localized AtCYPs

Screening a series of T-DNA insertion KO *Arabidopsis* mutants has disclosed that AtCYP40 plays intrinsic roles in the organogenesis of plants [20,37]. AtCYP40 is a unique multi-domain AtCYP, containing tetratricopeptide repeat domains that are able to bind ARGONAUTE 1 (AGO1) and HSP90 in the formation of an intermediate assembly of RNA-induced silencing complex (RISC) [39,40]. RISC is an effector complex of post-transcriptional gene silencing (PTGS), consisting of a single-stranded (ss) small RNA such as small interfering RNA (siRNA) and microRNA (miRNA) that is bound to an AGO family protein, which prevents the production of proteins from mRNAs that contain sequences complementary to the ss small RNAs, through cleavage or translational repression [41]. In this system, AtCYP40 promotes binding of AGO1 with a molecular chaperon, HSP90, to facilitate RISC assembly via an ATP-dependent chaperone cycle [40,42], which in turn stimulates the production of miRNAs (e.g., miR156) [39]. Constitutive expression of miR156 then prolongs the juvenile phase of vegetative development and increases the rate of leaf initiation [43,44,45]. Thus, *AtCYP40* KO mutant plants showed an alteration of leaf numbers, leading to a precocious expression of adult vegetative traits without induction of the reproductive maturation of shoots [20,39].

Two single-domain, AtCYP18-3 and AtCYP19-1 are highly homologous (95% aa sequence similarity) AtCYPs, but have displayed distinctive activates. Firstly, AtCYP18-3 is a multi-functional protein involved in plant growth, hormone signaling, and defense responses against biotic and abiotic stresses [46,47,48,49,50,51,52]. In the context of plant growth, AtCYP18-3 is positioned at the interface between light and brassinosteroid (BR) signaling pathways. BR signal antagonizes light-dependent seedling development, switching etiolation to de-etiolation by inhibiting cell elongation and promoting chloroplast development [23]. Hence, partial loss-of-function *AtCYP18-3* alleles displayed elevated sensitivity to BR in the light, which subsequently arrested de-etiolation processes (photomorphogenesis) [50]. On the other hand, AtCYP19-1 is considered to control seed development as the promoter trapping detected its expressions predominantly in the peripheral endosperm and in the late heart stage of embryo development [53]. However, earlier RNA blotting assays argued that AtCYP19-1 is expressed also in seedlings, stems and leaves of *Arabidopsis* [15], suggesting that it acts in diverse physiological functions beyond seed organogenesis (e.g., immune responses; see Section 3).

### 2.3. Chloroplast Localized AtCYPs

*Arabidopsis* chloroplasts include five AtCYPs in the thylakoid lumen (i.e., AtCYP20-2, AtCYP26-2, AtCYP28, AtCYP37 and AtCYP38) and one in the stroma (AtCYP20-3), of which AtCYP20-2 and AtCYP38 showed the functional involvements in the assembly and maintenance of photosystem (PS) components [54,55,56]. For instance, AtCYP20-2 showed a physical association with thylakoid membrane-embedded NAD(P)H dehydrogenase (NDH) complexes that mediates cyclic electron (e^−^) transport in photosynthesis, and chlororespiration [54,57]. In fact, the level accumulations of AtCYP20-2 were strongly reduced in *NDH*-defective mutant plants, suggesting that its functions as an auxiliary protein in the biogenesis of NDH complexes [54,58]. Besides, *AtCYP38*-deficient mutants exhibited significant reduction of the biogenesis and the half-life of PSII complexes, which in turn rendered PSII centers extremely susceptible to photoinhibition [55,59], indicating that AtCYP38 is necessary for the assembly of PSII and stabilization of light-dependent reactions of photosynthesis. It is worth nothing that a recent report has revealed that AtCYP20-2 can also bind and stimulate a BR signaling component, a BRASSINAZOLE RESISTNAT1 (BZR1) transcription factor (TF) in activating the expression of *FLOWERING LOCUS D* and promoting early flowering [56]. However, further studies are necessary to define (a) how plastidic AtCYP20-2 can interact with nuclear BZR1 TF, and (b) if expressions of *AtCYP20-2* are differentially regulated in leaf plastids (perhaps constitutive) vs. flowers (temporal)—if so, how?

### 2.4. Endoplasmic Reticulum (ER) Localized AtCYPs

Thus far, the TargetP has identified that five single-domain AtCYPs (i.e., AtCYP19-4, AtCYP20-1, AtCYP21-1, AtCYP21-2 and AtCYP23) are located to the endoplasmic reticulum (ER), a protein secretory pathway [1,7,60,61]. Indeed, the subcellular distribution of green fluorescence proteins fused with a signal peptide of AtCYP19-4 confirmed the ER localization of AtCYP19-4, especially in the apical cells of young stem and peduncle tissues [17,62,63], where it can physically bind a GNOM protein. GNOM is an ADP ribosylation factor-guanine-nucleotide exchange factor, that fine-tunes vesicular formations in membrane trafficking, and a cellular polarity along the apical-basal embryo axis control [17]. These interactions suggested that AtCYP19-4 may chaperone the activity of GNOM in the endosomal recycling of the auxin-efflux carrier PINFORMED1 to the basal plasma membrane in provascular cells, which in turn is required for the accumulation of the plant hormone auxin at the future apical meristems through polar auxin transport [17,63].

AtCYP20-1 belongs to a family of unfolded protein response (*UPR*) genes, responsive to ER stress [64]. In this context, a promoter region of AtCYP20-1 contains a X-box binding protein 1 (XBP1). During ER stresses, an ER transmembrane protein kinase/riboendonuclease (Ire 1p) is activated and splices the mRNA of XBP1. Matured XBP1 is then translocated to the nucleus where it binds to and activates the *cis*-acting element of *AtCYP20-1* [64,65]. Once expressed, AtCYP20-1 binds to PP2A, ubiquitous Ser/Thr protein phosphatase, that regulates multiple pathways in plant growth and defense responses [16,66]. In fact, T-DNA insertion KO mutant *Arabidopsis* of *AtCYP20-1* (*rcn1*) exhibited the drastic reduction of root and hypocotyl growth under ER stress, mimicked by toxic cantharidin treatments [16,67], suggesting that AtCYP20-1 play a critical role in proper protein synthesis and folding, as well as a removal of misfolded proteins during the life cycle of plants [68].

### 2.5. Mitochondria and Golgi Localized AtCYPs

Previously, two homologous AtCYP21-3 and AtCYP21-4 were predicted as mitochondrial AtCYPs [1,7]. A recent study, however, showed that AtCYP21-4 is likely localized at the Golgi apparatus [69] carrying out various post-translational modification processes including the glycosylation of proteins, producing glycoproteins [70,71]. In plants, glycoproteins play crucial roles in a variety of processes, e.g.,) forming cell wall matrixes, and optimizing morphogenesis under resting and stressed states [72,73]. Indeed, transgenic potatoes overexpressing *AtCYP21-4* demonstrated increased glycoprotein contents in all tissues, as well as higher yields (size and number of tubers), substantiating the intrinsic roles of AtCYP21-4 in plant growth and development via stimulating glycoprotein synthesis or glycan processing in the Golgi apparatus [69,74].

## 3. Roles of Cyclophilins in Defense Reponses against Abiotic Stresses

Environmental stresses such as heat, cold, drought, salt and excess water are major limiting factors in plant growth and productivity. As sessile organisms, plants employ elaborate regulatory pathways that rapidly rearrange the temporal and spatial profiles of gene expressions in responding and adapting those abiotic stresses [75,76,77]. Over recent decades, a large number of studies have utilized various transcriptome and bioinformatics analyses to delineate the genetic and functional circuitry of plant stress defense responses [78]. Of these studies, five *AtCYP* transcripts were found to be stress responsive; the heat shock-dependent induction of cytosolic *AtCYP18-1*, cold-dependent upregulation of plastidic *AtCYP19-2*, salt-dependent induction of cytosolic *AtCYP18-3* and ER *AtCYP19-4*, salt-responsive downregulation of cytosolic *AtCYP18-4* [7,79,80], but further investigations are needed to understand their roles in plant stress physiology.

Intrinsic activities of AtCYPs in the activation of plant stress response machineries have been further substantiated by the analyses of *Arabidopsis* KO mutant plants. For instance, disruption of *AtCYP20-3* and *AtCYP21-2* demonstrated enhanced hypersensitivity towards abiotic environmental stresses such as high light, oxidative, salt and/or water stresses [81,82]. Interestingly, expression of *AtCYP21-2* is highly upregulated during ER stresses that can be caused by various endogenous and exogenous stresses [64,65], suggesting that ER stress-responsive genes such as *UPR* genes play potentially important roles in a broad range of stress defense responses. However, the same states of ER stress did not induce the other *UPR, AtCYP20-1* (Section 2.4), discerning *AtCYP21-2* as a defense responsive gene while *AtCYP20-1* as plant growth regulators. Besides, ER stress showed little effect on *AtCYP19-4* transcripts, but salt stress caused the moderate level increases (~2-folds) in *AtCYP19-4* mRNA [7,82]. As alluded, AtCYP19-4 is involved in, unlike AtCYP21-2, the regulation of ER-mediated secretory system, perhaps explaining the need and roles of distinct metabolic pathways for the resolution processes of comparatively reverse stresses (i.e., salt/drought vs. excess water stresses). Note that AtCYP20-3 is the best-characterized AtCYP, and we will discuss its possible mode of actions during stressed and resting states in the Section 4 and Section 5.

## 4. Roles of Cyclophilins in Disease Resistance against Pathogen Infections

To understand the potential roles of AtCYPs in the plant and microbe interactions, two recent studies have carried out meta-analyses and found the activation of *AtCYP19-1* and *AtCYP57* expressions by the infection of pathogenic bacteria, *Pseudomonas syringae* and *Xanthomonas campestris* [83,84]. Pogorelko’s group [83] has then followed up to show that the disruption of *AtCYP19-1* and *AtCYP57* expressions enhance susceptibility, whereas the overexpression of *AtCYP19-1* and *AtCYP57* can promote disease resistance against *P. syringae* infections, providing solid evidence that AtCYP19-1 and AtCYP57 play intrinsic roles in the activation of immune responses. In parallel, they have utilized the yeast two-hybrid assays to probe the interactions of AtCYP19-1 with antioxidant regulators such as ENGD1 (GTPase/GTP-binding protein) and Rm1C like cupins [84], hypothesizing that the upregulation of *AtCYP19-1* expression is lined with the temporal modulation of antioxidant and detoxification systems to increase ROS accumulations shown in the *AtCYP19-1*-overexpression plants [83,85]. On the other hand, the overexpression of *AtCYP57* induced callose depositions, which is perhaps via binding and stimulating the activity of pyruvate decarboxylase I whose overexpression demonstrated increased callose depositions and expression of defense genes, in conjunction with anaerobic alcohol formation and soluble sugar formation [83,85,86].

Recently, emerging evidences have proposed that plants possess several restriction factors, being able to interfere with the viral replications by directly targeting viral replicase complexes (VRC) in the cytoplasm of infected cells [87,88]. Among the plant restriction factors are two cytosolic AtCYPs (i.e., AtCYP18-3 and AtCYP19-3), which showed binding affinity to the (+)-stranded RNA and/or replicase of tomato bushy stun tombusvirus (TBSV). These interactions then impeded the *de novo* replication of TBSV RNA via the inhibition of viral RNA recruitment, subsequently blocking the VRC assembly. In line with this scenario, the overexpression of *AtCYP18-3* and *AtCYP19-3* in plants, *Nicotiana benthamiana*, manifested the significant reduction of TBSV RNA accumulations, and the suppression of disease symptom development [51].

It is worth noting that AtCYP18-3 along with AtCYP18-4 and AtCYP20-3 were reported to interact with a virulence gene (VirD2) of *Agrobacterium tumefaciens*, a causative pathogen of a crown gall tumor disease on a wide variety of dicotyledonous plants by transporting a transfer (T)-DNA, a ss DNA segment of the tumor-inducing plasmid, from the bacterium to the plant cell [46,89,90]. These interactions hypothesized that AtCYPs are involved in maintaining the correct structural and/or functional states of VirD2. Indeed, incubation of *Arabidopsis* and tobacco cells with the CsA showed decreased T-DNA translocations, and perhaps disease establishment [46]. However, a recent finding by van Kregten et al. [91] that the VirD2 CYP-binding domain is not necessary for the T-DNA transformation suggests that CYPs may not be absolute requirement for VirD2 activity in the plant cells [90]. Alternatively, AtCYP18-3 may act as a negative regulator in defense activation, targeting and inhibiting the receptor (i.e., RPS2 and RPM1)-mediated recognition of pathogens (e.g., *P. syringae* DC3000 *avrRpt2*, *avrB* and *avrRpm1*). Therefore, the gain-of-function mutation of *AtCYP18-3* demonstrated the loss of receptor (collectively called resistance (*R*)-gene)-mediated disease resistance [52].

Lately, affinity screening has identified AtCYP20-3 as a signal receptor of plant defense hormone, (+)-12-oxo-phytodienoic acid (OPDA), belonging to jasmonate family of hormones which includes jasmonic acid, its precursors and derivatives [92,93]. OPDA is an autonomous signaling molecule that regulate unique subsets of jasmonate-responsive genes in activating and fine-tuning defense (adaptive) responses against necrotrophic fungi and insect herbivores, as well as growth processes [94,95]. When OPDA is produced under stress states, it binds and stimulates AtCYP20-3 to form a complex with serine actyltransferase1 (SAT1), which triggers the formation of a hetero-oligomeric Cys synthase complex (CSC) with *O*-actylserine(thiol)lyase B [92,96,97]. CSC formation then leads to the production of CYS (sulfur assimilation) and subsequently thiol metabolites, which increases cellular reduction potentials. The enhanced reduction capacity in turn coordinates the expression of a subset of OPDA-responsive genes that actuate and calibrate immune responses. Hence, the disruption of *AtCYP20-3* expression concurred with the enhanced disease susceptibility against necrotrophic fungal (e.g., *Alternaria brassicicola* and *Botrytis cinerea*) and oomycete (*Pythium irregulare*) pathogens [92,98].

## 5. Roles of Cyclophilin at the Interface between Plant Growth and Defense; A Case Study of AtCYP20-3

Emerging outcomes from a number of recent studies on AtCYPs have underpinned that CYPs are versatile metabolites in plants regulating various processes in growth and survival. In particular, AtCYP20-3 is found to be positioned within multiple signaling and metabolic pathways, binding with several interacting partners including SAT1, thioredoxins (Trxs) and 2-Cys peroxiredoxin (2-CysPrxs) in the chloroplasts [81,92,93,99,100,101], which propose AtCYP20-3 to be a key regulator in controlling the interface between OPDA (defense) and light-dependent redox (growth) signaling. The latter, also referred to as the electron (e^−^) transport chain (ETC) of PSI, is a primary metabolism converting solar energy into biologically useful chemical energies, which is a source of the overall biomass of plants and living organisms [93,102]. When PSI antenna captures solar energy, it excites e^−^ that reduces Trxs via a ferredoxin (Fd) and a Fd-Trx reductase. Trxs, small oxidoreductases, then delivers e^−^, and activates target enzymes in the Calvin cycle (carbon fixation) that balances consumption in photosynthesis [103,104,105]. Recent studies however have started to unveil that Trxs also target other, Calvin cycle-unrelated proteins, including AtCYP20-3 [106], a key regulator of (a) OPDA signaling (see above) and (b) photosynthetic pathway as an e^−^ donor of 2-CysPrxs which metabolize the detoxification of a toxic byproduct in photosynthesis (e.g., H_2_O_2_), and the activation of Calvin cycle enzymes [99,100,101,107]. The interaction of Trxs with AtCYP20-3, thereby, positioned AtCYP20-3 as a redox sensor of ETC, transferring e^−^ from Trxs towards 2-CysPrxs and/or SAT1. Reduction (activation) of 2-CysPrxs then continues peroxide detoxification and activates photosynthetic carbon metabolisms, whereas the activation of SAT1 stimulates sulfur assimilation which coordinates redox-resolved nucleus gene expressions in defense responses against biotic and abiotic stresses [92,108,109,110,111]. In line with this scenario, stress-induced OPDA binds and, perhaps, modulates the functional and conformational states of AtCYP20-3 to which adjusts its subsequent binding and electron transfer between 2-CysPrxs and/or SAT1 [92,93], hypothesizing that AtCYP20-3 is a unique player in controlling the interface between OPDA signaling and photosynthesis. This interplay thus enables plants to make an adaptive decision in allocating resources (e^−^) between growth and defense responses (e.g., fitness tradeoffs) towards constant environmental challenges such as pathogens, pests, tissue injury, as well as light and oxidative stresses [110,112,113]—in the end—ensuring optimal growth, reproduction and survival of plants.

## 6. Conclusions

Plants constantly cope with a vast array of environmental challenges whilst concurrently optimizing their fitness by reprogramming the growth and reproduction processes. Towards that, plants employ a number of primary and secondary metabolites, and intricate signaling network to interconnect and orchestrate multiple layers of complex cellular mechanisms. As discussed in this review, a growing number of studies have espied that plant CYPs are highly versatile protein regulators involved in a variety of metabolic signaling and pathway during plants growth and survival, suggesting that the activity or activities of each CYP and their functional crosstalk play intrinsic roles in controlling many of key regulatory hubs (e.g., AtCYP20-3) that coordinate the growth, development, as well as immune and defense responses in plants. Noticeable, CYPs are structurally conserved PPIases, and thus molecular components and mechanisms in which are involved their activities, likely share common ancestry and evolutionary processes across the plant Kingdom. Therefore, furthering our understanding of functional and biological activities between, and within plant CYPs will not only: (i) provide new insights into the cellular mechanisms that plants use to make adaptive decisions when challenged by multiple stressors; and (ii) can enrich plant breeding and engineering strategies for selection of elite genetic traits that will maximize plant fitness; but also (iii) assist understanding the immune activation of a mammalian system; and (iv) help improving drug developments through facilitating the rational design of more potent and safe reagents.
